# Social Correlates of HIV-Risky Behaviours among African Canadian Adolescents Living in British Columbia, Canada: A Secondary Data Analysis

**DOI:** 10.3390/ijerph20116031

**Published:** 2023-06-01

**Authors:** Emmanuela Nneamaka Ojukwu, Helen Uche Okoye, Elizabeth Saewyc

**Affiliations:** School of Nursing, University of British Columbia, Vancouver, BC V6T 2B5, Canada; helen.okoye@ubc.ca (H.U.O.); elizabeth.saewyc@ubc.ca (E.S.)

**Keywords:** African Canadian adolescents, HIV, risky behaviours, black youths, health disparity, social determinants of health, socio-ecological model

## Abstract

**Highlights:**

**What are the main findings?**

- African Canadian adolescents generally have low rates of HIV-risky behaviours;
- Rates increased from 2003 to 2018; particularly rates of condomless sex and sex due to the influence of drugs or alcohol;
- Of the sexually active in 2018, ≈50% had 2 or more partners and condomless sex at last intercourse;
- Unsafe neighbourhood, perceived racism and sexism were significant predictors of HIV-risky behavior.

**What are the implications of the main findings?**

- Engagement in sexual risky behaviours may expose African, Caribbean and Black adolescents to HIV-risk.
- Addressing the impacts of social determinants of health and socioecologic factors such as the absence of familial support, the negative influence of peer pressure (physical and sexual abuse, and exposure to substances) can help mitigate engagement in HIV-risky behaviors among African, Caribbean and Black adolescents.
- The provision of safe, stable housing as well as feeling safe in schools can help mitigate engagement in HIV-risky behaviors among African, Caribbean and Black adolescents.
- Racism, sexism and various forms of intersecting stigma must be sufficiently addressed to curb rates of engagement in HIV-risky behaviours among African, Caribbean and Black adolescents.

**Abstract:**

Studies have linked HIV-risky behaviours among young people to several socio-contextual factors. However, the social factors that might increase African Canadian adolescents’ exposure to HIV-risky behaviours, including unprotected sex and forced or multiple-sexual partnerships, have received little or no attention in the literature. Using data from the British Columbia Adolescent Health Surveys (2003–2018) and guided by intersectionality and socio-ecological frameworks, we examined the social determinants of HIV-risky behaviours (HRB) among African Canadian adolescents in British Columbia. We observed a general decline in HRB from 2008 to 2018. However, more than half (54.5%) of the 1042 who were sexually experienced in 2018 reported having 2 or more sexual partners, and nearly half reported condom-less sex. Our results demonstrate an important need to evaluate the impacts of several social factors on health outcomes for a unique, marginalized population.

## 1. Introduction

Health-harming behaviours such as unprotected sex, forced sex, sex with multiple partners, and injection drug use may emerge during adolescence [1,2] and can lead to exposure to sexually transmitted infections, such as the human immunodeficiency virus, (HIV) [1,3,4]. Of the 62,050 people living with HIV in Canada by the end of 2018, the majority of cases among persons aged 15 and older were through heterosexual contact [5]. Unprotected sexual intercourse among adolescents not only has repercussions for increased HIV-risk, but also teen pregnancy, which often has maternal, neonatal, social, and obstetrical detriments for adolescent girls [6,7], as well as delayed or incomplete formal education and economic consequences for boys [8].

Several social determinants of health have been associated with engagement in HIV-risky behaviours. Social determinants are defined as the conditions in which people are born, grow, work, live, and age, as well as the larger set of factors and institutions that shape their daily life circumstances [7]. Social determinants of health may present at the micro, meso, exo, or macro levels of the human ecological system [9] and exert influence on human behavior at the individual, relationship, community, and societal levels, according to the socio-ecologic model [10]. At the individual level, intrapersonal characteristics such as social identities and other socio-demographic characteristics may contribute to HIV-risky behaviours among adolescents. For example, identities that have been associated with an increased likelihood of HIV-risky behaviours include ethnic minority status, e.g., African, Caribbean, or Black [3,11], being gay, lesbian, or bisexual [12,13], and low socioeconomic status [4].

At the relationship or interpersonal level of the socio-ecologic model [10], interactions and relationships within people’s social networks influence their behaviours. Adolescence is a critical stage in the normative development of youth [14], when they begin to harness and explore their innate characteristics, aiming to discover their social identities and acquire a sense of belonging. However, negative influences from peers [15] and unequal power dynamics in sexual relationships may lead to acts that increase exposure to HIV. For example, gang involvement and violence exposure, including bullying, rape or forced sex, dating violence, assault, or abuse of any kind (physical or sexual), have been linked to increased risk for HIV exposure [2,16,17,18]. Further, adolescents might engage in unprotected sexual activity as a means to cope with the psychological distress they experience in their daily lives [19]. For instance, adolescents who are victims of sexual violence, such as forced sex, are more likely to further engage in HIV-risky behaviours [20]. On the other hand, some relationships protect against HIV-risky behaviours. Positive relationships adolescents may have with their parents, family, or peers, and support obtained through connectedness to schools or religious organizations [11,18,21], may help protect against engagement in HIV-risky behaviours. For example, [21] found that positive parenting was associated with a decreased likelihood of sexual risk. Additionally, neighbourhood quality had an indirect effect on sexual risk. Such quality might include a measure of safety, economic security, and/or access to amenities that are important for adolescents living in those neighbourhoods.

Lastly, community and societal-level factors identify larger social constructs that are pertinent when examining health among marginalized populations. Factors such as discrimination based on race, gender, and sexual orientation have been found to have detrimental effects on the health and well-being of adolescents [13,22,23]. A body of evidence in the United States [22,23,24,25,26] and Canada [13,27] suggests that adolescents of African descent who have experienced racism are more likely to report poorer health outcomes, which can have lasting effects, even in adulthood [22]. Particularly, a Canadian study suggests that experiences of racism have increased over the years among Canadian adolescents, and these experiences have been linked to detrimental health outcomes, such as higher odds of suicidal ideations and attempts, stress, substance use, and poorer emotional and mental well-being for these youths [27].

These risk and protective factors differ for ethnic minority adolescents [3,11,20], who are also disproportionately affected by HIV. In Canada, studies have focused either on a general population of adolescents or on other ethnic groups, such as Indigenous adolescents [13,28,29] or Asian Canadian adolescents [30]. As the African Canadian population remains severely understudied in the country, we decided to focus solely on this population for this manuscript, with the aim of uncovering factors that might be sources of health inequities and disparities suffered by this population. Future studies may then compare the sources of these disparities across races. However, to enable this process, knowledge must be garnered for each population, and we begin this process, bearing the outcome of our study in mind, by focusing on one of the populations most negatively affected and with a disproportionate rate of HIV incidence. Therefore, our study contributes to this knowledge gap by exploring the social correlates of HIV-risky behaviours among African Canadian adolescents in grades 7 to 12 from 2003 to 2018. Specifically, the study addressed the following research questions: (1) What are the differences in HIV-risky behaviours such as condom-less sex, forced sex, sex with multiple partners, etc., among African Canadian adolescents from 2003 to 2018? (2) What are the social factors at the individual, relationship, community, and societal levels of the socio-ecological model that predispose African Canadian adolescents to HIV-risky behaviours?

## 2. Theoretical Framework

**Figure 1:**
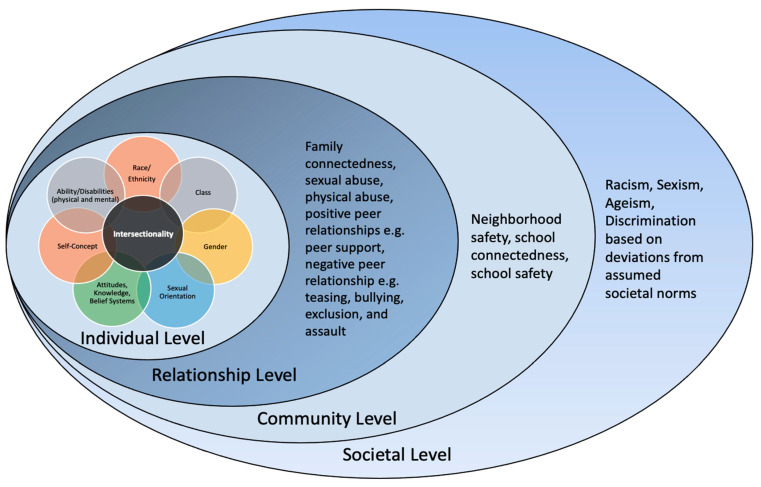
Adaptation of the socio-ecological model and intersectionality framework to engagement in HIV-risky behaviours among African Canadian adolescents.

This study was guided by the socio-ecological model [10] and the intersectionality framework [31]. This socio-ecological model [10], adapted from Bronfenbrenner’s ecological systems theory [9], describes the interrelatedness between people and their environment and how such interactions within the human ecosystem can influence behaviours. The intersectionality framework posits that individuals’ identities, such as race, gender, sexual orientation, socio-economic status, among others, act interdependently and synergistically to influence their behaviours and well-being. Taken together, these frameworks work synergistically to explain intersections in African Canadian adolescents’ identities (e.g., race, gender, sexual orientation, socio-economic status) at the micro-level and in their interactions with larger social constructs within the human ecosystem ranging from immediate familial influence to institutional (school) and societal contexts, which affect behaviour and health outcomes. In Figure 1, we provide our proposed conceptual model of how these two frameworks (intersectionality and the socio-ecological model) may work together to address the research question. The model shows the socio-ecological model as the overarching framework and demonstrates its interrelatedness with the intersecting identities at the individual level.

## 3. Methods

### 3.1. Survey Design

We used data collected from the British Columbia Adolescent Health Survey (BC AHS) to examine the differences in HIV-risky behaviours across survey years and the association between selected socio-ecological factors and HIV-risky behaviour. The BC AHS is a province-wide survey that uses a cluster-stratified sampling method to survey randomly selected classrooms and students in grades 7 through 12 from school districts across BC [12,32]. The survey procedures were approved by the Behavioural Research Ethics Board of the University of British Columbia (#H17-01307), and the first survey was conducted in 1992, then again in 1998, and has been repeated every 5 years since, with the most recent survey in 2018. The questionnaires used in these surveys have remained mostly the same to allow for comparability. The data obtained are weighted and scaled up to represent enrolled students within the province in each survey year.

### 3.2. Sample

The current study focused on data from the BC AHS of 2003, 2008, 2013, and 2018. We could not include the surveys prior to 2003 because the datasets did not contain a measure of ethnicity for African Canadian adolescents in British Columbia.

To ensure consistent findings across the survey years, we selected the school districts that participated in at least three out of the four survey years. These schools represented 94.2% of all enrolled students in the selected survey years.

### 3.3. Inclusion and Exclusion Criteria

We included students who self-identified as (1) Africans and (2) self-reported ever having had sex. To ensure consistent findings across the survey years, we excluded school districts that did not participate in at least three out of the four survey years (2003–2018).

### 3.4. Measures

*Dependent Variable.* Our dependent variable was HIV-risky behaviours, conceptually defined as behaviours common among adolescents that predispose or expose them to HIV-risk, such as unprotected wanted or unwanted sexual encounters, multiple-partner sexual encounters, and/or sexual encounters impaled by substance use, among others [2,29]. HIV-risky behaviour was created from eight variables. These were: unwanted sexual intercourse under the influence of drugs or alcohol; intentional alcohol or drug use at last sexual intercourse; past year number of sexual partners; ever been pregnant or ever caused a pregnancy (which we labelled “pregnancy involvement”); having been forced to have sexual intercourse by an adult; having been forced to have sexual intercourse by another youth; no condom use at last sex; and ever been diagnosed with a sexually transmitted infection. The selection of these indicators was informed by their associations with HIV-risk among young people in the literature [2,16,17,18,29]. All the selected variables had binary responses (yes or no) in the dataset, except the number of sexual partners and pregnancy involvement. We dichotomized the number of sexual partners as “one or less” versus “2 or more” sexual partners, consistent with the literature that a higher number of sexual partners increases HIV-risk [16,33]. Similarly, the number of times an adolescent had “ever been pregnant or ever caused a pregnancy” was dichotomized as “never or zero times” versus “one or more times”. From the summative score of all 8 HIV-risky behaviours indicated above, an HIV-risk index was created for a total of 8 points. Based on a population mean of 2.4/8 among all indicated HIV-risky behaviours and through iterative conversations among the authors, a cutoff of 3 or less was indicated as low risk, and values greater than 3 were indicative of moderate-high risk. Thus, HIV-risky behaviours were dichotomized as “low risk” (those who scored 0–3 out of all 8 total points, coded as 0) versus “moderate to high risk” (those who scored 4–8 out of 8 total points, coded as 1).

*Independent Variables.* For the analyses of social determinants of health with the 2018 BC AHS, we explored variables at different levels of the socio-ecological model [10] and their associations with HIV-risky behaviour, either as risks or protective factors. At the individual level, we examined variables such as adolescents’ age, assigned sex at birth, birthplace in Canada or not, problematic levels of alcohol or drug use (measured by adolescents who reported episodes of passing out after using drugs or alcohol), and lifetime frequency of illicit drug use (cocaine, heroin, ecstasy, mushrooms, etc.). We also examined adolescents’ self-report of their mental health, which was a continuous variable that asked adolescents to rate their mental health from poor to excellent (rated as 1–4) [4]. We also examined other mental health challenges, such as feeling strained, stressed, or pressured in the last 30 days and how they manage stress and pressure, as well as questions about suicidal ideations and attempts in the past year. Most of these variables were originally measured as binary or recoded from ordinal measures and grouped into fewer relevant categories. We included a variable that assessed how many times teens went to bed hungry as a proxy for poverty. Possible responses were never, rarely, or often/always. We also measured binge drinking based on recoding a single question: during the past 30 days, how many days did you have 5 or more drinks of alcohol within a couple hours (zero number of days, 5 or fewer days, and more than 5 days).

At the interpersonal level, we reasoned that positive family relationships might be protective: these included with whom adolescents lived most of the time, if adolescents had an adult confidant (if they had any adult within their family with whom they could rely on for help), and family connectedness. We used the family connectedness scale included in the survey [34], which included questions such as, “How much do you feel that people in your family understand you? Pays attention to you? You and your family have fun together?” Other questions measured potential risks: we examined physical abuse from either family or peers with a single “yes” or “no” question (Have you ever been physically abused by anyone in your family or anyone else?). Further, we examined the impact of sexual abuse from family and peers with a similar question. In addition to physical and sexual abuse from peers, more negative peer experiences, such as dating violence and being teased, excluded, or assaulted at school or on their way to and from school in the past 12 months (which was a “yes” or ‘no” question), were also examined. In evaluating the impact of positive peer relationships, we examined if adolescents could count on help from their friends (online or in person) if they had a serious problem.

We also included community-level protective factors, such as school safety, school connectedness, and neighbourhood safety. School connectedness and school safety were existing scales created from multiple items that assessed adolescents’ feelings of school belonging and teacher or staff caring and the level of feeling safe in various locations around the school [35]. These factors have also been connected to adolescent health outcomes in Canada [34]. Neighbourhood safety assessed the extent to which African Canadian adolescents felt safe during the day or at night in their neighbourhood. At the societal level, we examined experiences of racism, sexism, ageism, or discrimination based on physical appearance, and potential associations with HIV-risky behaviour. These variables were all measured dichotomously.

### 3.5. Data Analyses

We carried out a secondary data analysis using the Complex Samples Module in IBM SPSS for windows version 28.0, IBM Corp, New York, NY, USA) to adjust for oversampling and the cluster-stratified sampling design that was used in the original survey. Descriptive statistics, including frequencies, percentages, means, and standard errors, described the sample characteristics. We examined differences across survey years for each HIV-risky behavior across the four survey years (2003, 2008, 2013, and 2018). For the analyses of social correlates of HIV-risky behaviour, we focused on the 2018 survey only due to the need to present the most recent status quo on HIV-risky behaviours within this community. To examine the social factors that influenced HIV-risky behaviour, we conducted a series of analyses. First, we conducted cross-tab analyses for all independent variables against our outcome variables to determine the association between the social factors and measures of HIV-risky behaviour. The independent variables, which were significantly associated with low and moderate-to-high groups from the crosstabulation analysis, were further analyzed using bivariate logistic regressions. Subsequently, we entered variables significant at the bivariate level into a multivariate logistic regression model to ascertain the social factors that had an overarching influence on HIV-risky behaviour. Considering the maturational effects on sexual behaviours (i.e., adolescents are more likely to engage in sex as they mature), we included age as a covariate in logistic regression analyses. All analyses were set at the 0.05 level of significance.

## 4. Results

### 4.1. Characteristics of African Canadian Adolescents from 2003 to 2018

The total weighted and scaled sample of African Canadian adolescents was 21,834, ranging from 4650 in 2003 to 5743 in 2018 (Table 1). Of these, the number of African adolescents who were sexually experienced ranged from 1072 in 2003, 1677 in 2008, 1331 in 2013, and 1111 in 2018 (Table 1). African Canadian adolescents in 2018 who had ever had sex were slightly older in age (*M* = 16.24) compared to other survey years. A majority of the adolescents were born in Canada, and the sample consisted of more boys than girls across the four survey years. In 2018, a majority of the adolescents (85.8%) had not reported poverty, and most spoke English at home across all 4 survey years. Almost all the teens were heterosexual or unsure of their sexual orientation, and a range of 3.2–16.2 percent said that they were bisexual, pansexual, gay, or lesbian.

### 4.2. Differences in HIV-Risky Behavior among African Canadian Adolescents across Survey Years from 2003 to 2018

**Figure 2:**
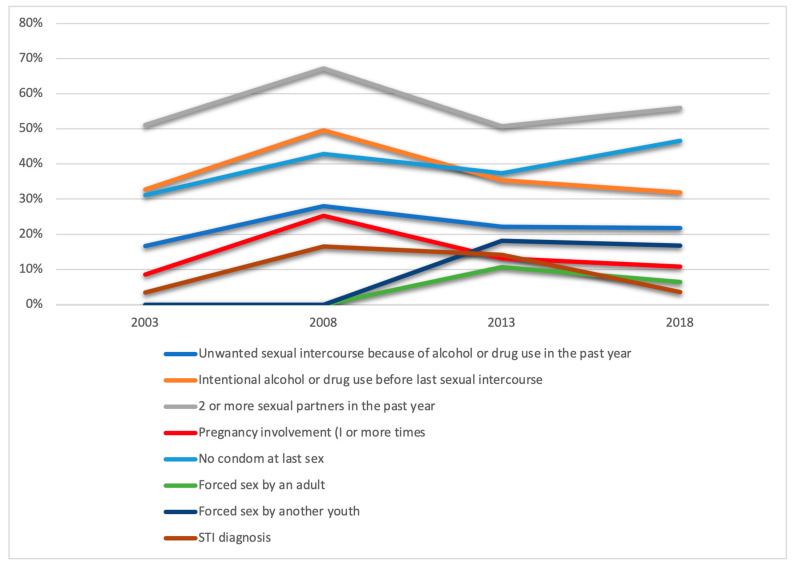
HIV-risky behaviours among African Canadian adolescents who have ever had sex from 2003 to 2018.

The Rao–Scott adjusted Chi-square analyses showed significant differences in the incidences of HIV-risky behaviour across survey years for sexually experienced African Canadian adolescents (Table 2; Figure 2). The highest incidence of risky behaviour across all four survey years was having two or more sexual partners and intentional alcohol or drug use before sexual intercourse. Both of these behaviours increased from 2003 to 2008, then decreased between 2008 and 2013, and increased again to their 2003 level or higher in 2018. These differences in years were statistically significant at *p* < 0.05. Unwanted sexual intercourse due to the influence of drugs or alcohol, intentional alcohol or drug use before sexual intercourse, and self-report of having been diagnosed with a sexually transmitted infection had all increased by approximately 70%, 52%, and 400%, respectively, from 2003 to 2008. All three behaviours then declined steadily from 2008 to 2013. From 2013 to 2018, the rates further declined, except for unwanted sexual intercourse due to the influence of drugs or alcohol. However, the noted differences in the rates for this variable (unwanted sexual intercourse because of alcohol or drug use in the past year) across all 4 years were not found to be statistically significant at *p* < 0.05. Other risky behaviours, such as forced sex by an adult or youth, which were not measured in 2003 and 2008, showed a general decline between 2013 and 2018. Forced sex by an adult decreased by almost half (46%), from 10.7% (2013) to 5.8% (2018), while forced sex by another youth decreased by only 1.1%, from 18.1% (2013) to 17.9% (2018). However, these differences in forced sex by an adult and by a youth were not found to be statistically significant at *p* < 0.05. 

### 4.3. Incidence of HIV-Risky Behavior among African Canadian Adolescents in 2018

Due to a need to present the most recent status quo on HIV-risky behaviours within the community, we focused on the latest survey from 2018 to examine the incidence of HIV-risky behaviours among all African Canadian adolescents. Of the estimated 5019 African Canadian adolescents represented in the 2018 survey, an estimated 1111 had reported ever having sex, and among those, 20.8% engaged in medium-high HIV-risky behaviour. We found that more than half had reported having two or more sexual partners, and nearly half did not use a condom the last time they had sexual intercourse. Intentional alcohol and drug use at the last sexual intercourse followed in third place, with 29.3% reporting this act, followed by unwanted sexual intercourse because of alcohol or drug use at 23.3% and forced sex by another youth at 17.9% (Table 3).

### 4.4. Social Correlates of HIV-Risky Behaviours among African Canadian Adolescents in 2018

The variables entered into the multivariate model (Table 4) were: adolescent self-reported mental health status; suicidal ideation; passing out from using alcohol or drugs in the past year; illicit drug use; being teased, excluded, and/or assaulted; physical abuse; sexual abuse; inability to say “no” to sex; neighbourhood safety; family connectedness; school safety and connectedness; racial discrimination; and gender. The variables entered into the model were significant during the bivariate analysis. In Table 4, we provide both the unadjusted odds ratio (OR) and the age-adjusted odds ratio (AOR) of the relationship between aspects of the social environment and engagement in health-compromising behaviours.

Upon analysis, the multivariate regression model was significant at *p* < 0.001. The model explained 54.1% (Nagelkerke *R*^2^) of the variance in HIV-risky behaviour and had an overall prediction accuracy of 88.8% of cases. The model showed that, controlling for all other variables, six variables remained statistically significant in predicting moderate to high HIV-risk (Table 4). Adolescents who passed out after using alcohol or drugs had 4.7 times higher odds of moderate to high HIV-risk (*p* = 0.008); adolescents who used illicit drugs 2 or more times in their lifetime compared to no use at all had approximately 9 times higher odds of moderate to high HIV-risk (*p* = 0.002); adolescents who could not say “no” to sex from a new partner, a long-term partner, or to sending nudes had 14 times higher odds of moderate to high HIV-risk (*p* = 0.009); and those who experienced racial and gender discrimination had approximately 4 times (*p* < 0.05), and 6 times (*p* < 0.05) higher odds of HIV-risk, respectively. Further, results showed that for every 1-point increase in neighbourhood safety, there were 54% (*p* = 0.003) lower odds of HIV risk.

## 5. Discussion

In Canada, persons who migrated from HIV-endemic countries, mostly African and Caribbean nations, have an HIV incidence rate 9.2 times that of those born in Canada or other migrants from non-endemic countries [36]. African (black) Canadians make up only 2.2% of the 34 million population, yet account for as much as 25.5% of HIV infection rates from heterosexual contact [5]. Despite a disproportionate level of HIV-risk behaviours and behaviours that result in pregnancy noted among racial minorities [3,11,17,18], there is limited research among black Canadian adolescents. Most studies examining these trends and associated risks or protective factors have been conducted in the United States [3,11,17,18] or in sub-Saharan African countries [4,37]. Notable Canadian. Therefore, with most other studies originating from African countries [37,38,39] or the US [16,18,25] and only one study in 2007 focused on African Canadian adolescents [40], our study fills a major gap by contributing evidence on this public health concern for a growing visible minority within the Canadian population. Consistent with the socio-ecological model, our findings reveal important social factors that expose African Canadian adolescents to HIV-risky behaviour. In analyzing differences in HIV-risky behaviours across survey years and the social correlates, our study calls attention to factors that may be contributing to the disproportionate rate of HIV infection among this population in Canada.

While our cross-sectional data analysis does not establish a causal relationship, descriptive and associative analyses showed that a majority of the adolescents in our study engaged in low-risk behaviours. There were also some concerning rates among sexually experienced youth in the most recent survey, 2018. Among these youths, nearly half reported condom-less sex, and more than half reported having two or more sexual partners. These rates were closely followed by intentional or unintentional sex under the influence of alcohol or drugs and forced sex by youth. These findings highlight higher frequencies of engagement in risky sexual behaviours and the negative influence of alcohol or drugs on sexual experiences. Experiencing forced sex is a high-risk sexual behaviour because such sexual encounters may not allow for condom negotiation and adequate protection. Forced sex has been associated with condom-less sex, sex with multiple partners, and sex while intoxicated [41], all of which can increase HIV risk.

In examining differences in HIV-risky behaviours across survey years, our results showed varying rates of alcohol or drug use before sex and unchanged rates of condom use among adolescents over the 15 years of surveys. This is concerning and begs the question of adequate HIV-risky-related knowledge and knowledge of alcohol and drug use’s influence on self-efficacy and confidence in negotiating condom use. Education efforts to promote safer sexual practices could be centred on peer influences, the harmful effects of substances on sexual decisions, and increasing self-efficacy and confidence in negotiating and using condoms, among other barriers. Given the percentage of adolescents in our study reporting going to bed hungry, which was a proxy measure for socio-economic status in our study, access to free or low-cost condoms may also be an important consideration.

Many of the youth reported using alcohol or drugs before sexual intercourse. When teens are in an impaired state under the influence of alcohol or drugs, they may be unable to negotiate condom use. Some studies have noted associations between condom-less sex and multi-partner sex and alcohol and drug use among youths [29,42]. Further, youths who had suicidal ideations had a higher likelihood of engaging in moderate-to-high HIV-risky behaviour. When adolescents experience traumatic behaviours or experience stress, they might become depressed and consider suicide. The literature corroborates this finding, as depression and suicidal attempts have been noted to have significant relationships with involvement in HIV-risky behaviours [39,43].

Not surprisingly, negative peer experiences such as being teased, excluded, or assaulted were significant predictors of moderate-to-high HIV-risk. Peer relationships and their influence on adolescent behaviours have received significant attention in recent years. Of note, the behaviours examined in the study—teasing, exclusion, and assault—are capable of causing social isolation, and evidence indicates that prosocial peers are protective against risky behaviours, while friendships with peers who engage in risky behaviours are likely to lead to similar actions [15].

Our study also found that adolescents who could not say “no” to sex with a new or long-term partner or to sending nudes had 14 times greater odds of engaging in HIV-risky behaviours. The reasons adolescents are unable to say “no” to sex may stem from reasons such as a mere enjoyment of sex or an inability to say no due to sexual coercion. Evidence from the literature suggests that the latter may be the case among the African female population. Several studies have alluded to young women’s inability to say no to unwanted sex stemming from dominant coercive partners [44,45], hegemonic norms of masculinity [46] and women’s normative ideas of men’s sexuality as violent, forceful, and brash, stemming from their experiences of such sexual coercion [45]. For dating women, several studies conducted with African women have pointed to a reduced agency in making reproductive decisions and a limited ability to exercise agency in sexual activities, leaving such women, unfortunately, vulnerable to sexual coercion and even sexual abuse [45]. Some studies report women giving in to sexual coercion for fear that their partner will force them if they refuse [44] and other studies report South African men conforming to dominant norms of masculinity in their sexual behaviours, often associated with increased HIV risk, though they give instances of efforts taken to protect themselves and their partners [46]. Surprisingly, sexual abuse in this study was not a significant predictor of moderate to high HIV-risky behaviour, although it approached significance at *p* = 0.07. However, these studies provide evidence and support our finding that sexism is a significant predictor of HIV-risky behaviour. Needless to say, our study and many others continue to lend voice to a call for action to educate African adolescent boys and girls about their reproductive rights and the agency of their bodies.

At the community level, we noted that perceptions of neighbourhood safety predicted lower odds of engaging in HIV-risky behaviours. Neighbourhood safety has been associated with risky behaviours sucalcohol and drugs [38]. Additionally, racialized people, who are often handicapped by limited resources, may be forced to segregate and live in certain neighbourhoods. Such segregation and concentration, often within limited infrastructure, may lead to impoverished neighbourhoods, which may force their inhabitants to consider other means of survival and generating income, including drug involvement, sexual work, etc., the consequence of which is an increased risk of violence and exposure to sexually transmitted infections, particularly HIV [47]. Studies of African adolescents residing in Africa have noted associations between unsafe neighbourhoods and a greater likelihood of substance use (alcohol and drugs). Notably, our study also found concerning rates of substance use among adolescents, with significant differences in proportions between the low versus the moderate to high HIV-risky groups.

We were surprised that family connectedness, school safety, and school connectedness were not significant protective factors for HIV-risky behaviours in the multivariate model, although they were all significant protective factors during bivariate analyses. It may be that experiences of racism and sexism, which remained significant predictors of HIV risk, permeate through the school systems, attenuating the effects of school connectedness and safety in the presence of racism and sexism in the same model. The relatively small sample size within a complex-sampled study design may have reduced the power to detect the effects of the variables when included in a model. Evidence abounds in the literature of the positive influences of family connectedness, school connectedness, and school safety on prosocial behaviours for adolescent boys and girls, so this finding warrants further exploration with other studies.

At the societal level, racial and gender discrimination resulted in higher odds of engaging in HIV-risky behaviours. It is undeniably evident in the literature that these forms of societal prejudice and bias can negatively impact health. Recent findings also suggest increasing rates of racism among African Canadian adolescents [27] and the resultant negative effect of racial and sexual orientation discrimination on health outcomes [12,13,27].

## 6. Strengths and Limitations

To our knowledge, our study remains the first of its kind to examine and contribute significant knowledge related to engagement in HIV-risky behaviours among a visible minority group that has remained understudied in British Columbia, one of Canada’s provinces known for its multiculturalism and diversity. We acknowledge that the British Columbia Adolescent Health Survey is self-reported data that is subject to social desirability. Thus, there is a need for caution in interpreting the results. However, our findings contribute evidence about the social factors that might increase African Canadian adolescents’ HIV-risky behaviour. Further, we acknowledge that heterogeneity exists even within the African/Black culture, and provincial laws and factors may influence behaviours as adolescents acculturate to their new immigrant homes. Therefore, African Canadian youths in British Columbia might differ from similar African Canadian youth in other provinces; hence, future studies may conduct similar analyses for African Canadian adolescents from other Canadian provinces. The results from these studies and ours may then be evaluated for cohesiveness, similarities, and/or differences. Additionally, future studies may consider cohort and longitudinal analyses to ensure temporality in support of more statistical predictions.

## 7. Patient and Public Opinion

To reduce the effects of cultural heterogeneity and in our efforts to ensure the reliability and validity of our results, we worked closely with an advisory group that consisted of persons from different African countries resident in Canada, including adolescents, healthcare providers, educational leaders, organizations, policymakers, and other knowledge users/providers, with an intent to provide feedback on the study and an opportunity to highlight and discuss any heterogeneity in black/African culture across multiple ethnicities that may hinder the generalizability of the study’s findings. The advisory group responded positively to the results of the study and acknowledged its importance. Ideas and recommendations for future studies were also shared.

## 8. Healthcare Implications

This study has implications for clinical education and practice. Healthcare providers are strategically positioned to provide information and education about HIV and work with families, schools, and governmental and nongovernmental agencies working in the area of HIV prevention to address related factors that predispose African Canadian adolescents to HIV-risky behaviours.

Within colleges and universities, student-physicians and nurses, among others, should be educated on factors and social factors that might increase HIV-risky behaviours among African Canadians. This subject matter may be provided during lectures, clinicals, or health fairs organized by the university. Such knowledge would be crucial in helping them identify vulnerable adolescents and intervene accordingly. African adolescent students may also benefit from sex education provided within their schools. In practice, healthcare providers should recognize the importance of screening African Canadian adolescents for their involvement in sexual practices or other HIV-risky behaviours. Concerning numbers of young African adolescents are engaging in HIV-risky behaviours, with some reporting a very early age of sexual debut in our study, even as early as 12 years old. This age group of adolescents might be assumed to be sexually abstinent or naïve, and studies have shown a reluctance for parents to discuss sexual risk with their teens [48]. Further, educators, policymakers, nurses, and all healthcare providers must be cognizant of societal biases and aim to provide empathetic, equitable care to this population.

## 9. Conclusions

Our study lends itself to timely intervention among recent calls for health equity in the public health system. Our study provides results of engagement in HIV-risky behaviour across a total of 15 years (2003–2018) among African Canadian adolescents, an important, growing population that has received very little research attention. To our knowledge, only one other study in Canada has focused on this population. The limited research, including this study, demonstrates a critical need to fill such gaps in order to address health disparities or inequities and their associated social determinants of health.

## Figures and Tables

**Table 1:** Sociodemographic characteristics of African Canadian adolescents who have ever had sex.

Sociodemographic Characteristics	2003 (*n* ^ = 1072)	2008 (*n* ^ = 1677)	2013 (*n* ^ = 1331)	2018 (*n* ^ = 1111)
Mean age	16.20	15.88	16.15	16.24
Gender				
Male	59.9%	65.3%	56.0%	64.7%
Born in Canada				
Yes	81.4%	71.1%	68.7%	65.4%
Spoke a language other than English at Home				
Never	61.%	51.0%	58.6%	44.3%
Sometimes	30.0%	28.5%	23.6%	37.9%
Most of the time	8.9%	20.5%	17.9%	17.2%
Going to bed hungry				
Never	^a^	^a^	70.9%	85.8%
Sometimes	^a^	^a^	13.8%	12.2%
Often/always	^a^	^a^	15.3	2.0%
Sexual orientation				
Heterosexual, no attraction, unsure, or questioning	89.9%	70.3%	81.6%	86.2%
Mostly Straight	7.0%	13.5%	7.8%	3.9%
Bi, pan sexual, gay, or lesbian	3.2%	16.2%	10.6%	9.9%

^ = weighted *n* ^a^ Not measured in these survey years.

**Table 2:** Trends in HIV-risky behaviour among African Canadian adolescents who have ever had sex.

Variable	2003 (*n* ^ = 1072)		2008 (*n* ^ = 1677)		2013 (*n* ^ = 1331)		2018 (*n* ^ = 1111)	
	%	CI	%	CI	%	CI	%	CI
Unwanted sexual intercourse because of alcohol or drug use in the past year	16.6%	(13.7–20.1)	28.0%	(21.6–35.5)	22.1%	(15.3–30.8)	23.3%	(17.7–29.9)
Intentional alcohol or drug use before last sexual intercourse ***	32.7%	(25.9–40.3)	49.6%	(42.2–56.9)	35.3%	(27.5–43.9)	29.3%	(22.8–36.8)
2 or more sexual partners in the past year **	51.1%	(45.1–57.0)	67.2%	(59.6–74.0)	50.7%	(42.8–58.7)	54.2%	(46.1–62.0)
Pregnancy involvement (I or more times ***	8.5%	(6.1–11.6)	25.3%	(18.1–34.2)	13.2%	(8.6–19.9)	9.3%	(5.7–14.7)
No condom at last sex	31.1%	(20.8–43.7)	42.8%	(36.6–49.2)	37.3%	(29.6–45.7)	48.4%	(41.2–55.6)
Forced sex by an adult	^a^		^a^		10.7%	(6.5–16.9)	5.8%	(2.9–11.0)
Forced sex by another youth	^a^		^a^		18.1%	(12.5–25.5)	17.9%	(12.9–24.3)
STI diagnosis ***	3.4%	(2.1–5.3)	16.5%	(11.8–22.5)	14.2%	(8.8–22)	3.4%	(1.8–6.5)

** *p* < 0.1 *** *p* < 0.001, ^ weighted *n,* ^a^ Not measured in the given survey year.

**Table 3:** Incidence of HIV-risky behaviours (HRB) among African Canadian adolescents who have ever had sex in 2018.

HIV-Risky Behaviours	Males (*n*)	Females (*n*)	Total % of Population Engaging in HIV Risky Behaviours
Unwanted sexual intercourse because of alcohol or drug use in the past	133	116	23.3
Intentional alcohol or drug use at last sexual intercourse	192	128	29.3
2 or more sexual partners	391	189	54.2
Pregnancy involvement 1 or more times	71	26	9.3
No condom at last sex	290	231	48.4
Forced sex by an adult	38	24	5.8
Forced sex by another youth	57	135	17.9
STI diagnosis	29	8	3.4

**Table 4:** A logistic regression of the social correlates of HIV-risky behaviours among adolescents who have ever had sex in 2018.

	Moderate to High-Risk HIV Behaviour			
	Unadjusted OR		Age-Adjusted OR	
**Individual level**				
Self-report of mental health	0.32	(0.13, 0.79) *	1.81	(0.89, 3.66)
Suicidal ideation in the past year	2.90	(1.35, 6.23) **	1.43	(0.37, 5.54)
Passed out because of using drugs or alcohol in the past year	5.79	(2.29, 14.67) ***	4.65	(1.50, 14.4) **
Illicit drug use (e.g cocaine, heroin, ecstasy, mushrooms, etc.) lifetime	4.93	(0.61, 2.11)	4.22	(1.95, 9.16)
2 or more times	4.93	(2.11, 11.52) **	8.89	(2.22, 35.45) **
1 or 2 times	0.46	(0.09, 2.41)	0.43	(0.06, 2.84)
0 times	Ref	-	Ref	-
**Relationship level**				
Negative peer experience (being teased, excluded, and/or assaulted)	2.69	(1.05, 6.85) *	1.30	(0.43, 3.96)
Physical abuse	2.48	(1.11, 5.56) *	0.32	(0.025, 4.16)
Sexual abuse	5.90	(2.56, 13.63) ***	7.77	(0.84, 71.65) ^#^
Could not say “no” to sex	4.3	(1.47, 12.68) **	14.06	(1.98, 99.99) **
**Community level**				
Neighbourhood safety	0.56	(0.39, 0.81) **	0.46	(0.28, 0.76) **
Family connectedness	0.73	(0.47, 1.12)	0.96	(0.52, 1.78)
School Safety	0.48	(0.32, 0.71) ***	1.79	(0.87, 3.70)
School connectedness	0.49	(0.271–0.87)*	0.64	(0.34, 1.22)
**Societal Level**				
Racial discrimination	3.13	(1.2, 8.16) *	3.95	(1.33, 11.73) *
Gender discrimination	3.42	(1.26, 9.28) *	6.39	(1.33, 30.75) *

**^#^** *p* = 0.07 * *p* < 0.05 ** *p* < 0.1 *** *p* < 0.001.

## Data Availability

The data that support the findings of this study are only available on request from the McCreary Centre Society. Restrictions apply to the availability of the specific subset of data which were used with permission for this study.
